# Modulation of Autophagy Influences the Function and Survival of Human Pancreatic Beta Cells Under Endoplasmic Reticulum Stress Conditions and in Type 2 Diabetes

**DOI:** 10.3389/fendo.2019.00052

**Published:** 2019-02-26

**Authors:** M. Bugliani, S. Mossuto, F. Grano, M. Suleiman, L. Marselli, U. Boggi, P. De Simone, D. L. Eizirik, M. Cnop, P. Marchetti, V. De Tata

**Affiliations:** ^1^Department of Clinical and Experimental Medicine, University of Pisa, Pisa, Italy; ^2^Department of Surgical Pathology, Medicine, Molecular and Critical Area, University of Pisa, Pisa, Italy; ^3^Department of Translational Research and New Technologies in Medicine and Surgery, University of Pisa, Pisa, Italy; ^4^Medical Faculty, ULB Center for Diabetes Research, Université Libre de Bruxelles, Brussels, Belgium

**Keywords:** autophagy, human islets, type 2 diabetes, ER stress, insulin secretion, beta cell dysfunction

## Abstract

Autophagy is the major mechanism involved in degradation and recycling of intracellular components, and its alterations have been proposed to cause beta cell dysfunction. In this study, we explored the effects of autophagy modulation in human islets under conditions associated to endoplasmic reticulum (ER) stress. Human pancreatic islets were isolated by enzymatic digestion and density gradient purification from pancreatic samples of non-diabetic (ND; *n* = 17; age 65 ± 21 years; gender: 5 M/12 F; BMI 23.4 ± 3.3 kg/m^2^) and T2D (*n* = 9; age 76 ± 6 years; 4 M/5 F; gender: BMI 25.4 ± 3.7 kg/m^2^) organ donors. Nine ND organ donors were treated for hypertension and 1 for both hypertension and hypercholesterolemia. T2D organ donors were treated with metformin (1), oral hypoglycemic agents (2), diet + oral hypoglycemic agents (3), insulin (3) or insulin plus metformin (3) as for antidiabetic therapy and, of these, 3 were treated also for hypertension and 6 for both hypertension and hypercholesterolemia. Two days after isolation, they were cultured for 1–5 days with 10 ng/ml rapamycin (autophagy inducer), 5 mM 3-methyladenine or 1.0 nM concanamycin-A (autophagy blockers), either in the presence or not of metabolic (0.5 mM palmitate) or chemical (0.1 ng/ml brefeldin A) ER stressors. In ND islets palmitate exposure induced a 4 to 5-fold increase of beta cell apoptosis, which was significantly prevented by rapamycin and exacerbated by 3-MA. Similar results were observed with brefeldin treatment. Glucose-stimulated insulin secretion from ND islets was reduced by palmitate (−40 to 50%) and brefeldin (−60 to 70%), and rapamycin counteracted palmitate, but not brefeldin, cytotoxic actions. Both palmitate and brefeldin induced PERK, CHOP and BiP gene expression, which was partially, but significantly prevented by rapamycin. With T2D islets, rapamycin alone reduced the amount of p62, an autophagy receptor that accumulates in cells when macroautophagy is inhibited. Compared to untreated T2D cells, rapamycin-exposed diabetic islets showed improved insulin secretion, reduced proportion of beta cells showing signs of apoptosis and better preserved insulin granules, mitochondria and ER ultrastructure; this was associated with significant reduction of PERK, CHOP and BiP gene expression. This study emphasizes the importance of autophagy modulation in human beta cell function and survival, particularly in situations of ER stress. Tuning autophagy could be a tool for beta cell protection.

## Introduction

Autophagy represents a highly conserved intracellular recycling pathway by which cellular components are degraded through the lysosomal machinery (3). Classically considered as a mechanism to promote cell survival during starvation (2), autophagy can also be induced by several physiological and pathological conditions, such as growth factors deprivation, hypoxia, oxidative stress, and physical exercise (4). Furthermore, autophagy seems to be constitutively activated at low levels to remove misfolded proteins and damaged and/or senescent organelles (1). Hence, autophagy can be viewed as a mechanism to protect cells against several stressors as well as a cellular response to wear-and-tear processes (5). On the other hand, it has been demonstrated that dysregulated activation of autophagy can also induce different types of cell death (6). Thus, it seems that autophagy can either protect or promote cell death in relation to the cellular and environmental context (7). Accordingly, altered autophagy could play a key pathogenic role in several disease processes, especially where the accumulation of damaged molecules and organelles might elicit a condition of increased cellular stress (8).

Pancreatic beta cells are specialized to secrete insulin in response to variations in blood glucose concentration. In order to maintain glucose homeostasis, beta cells are able to raise several fold their insulin synthesis and secretion in response to increased plasma glucose levels. Therefore, they must continually deal with a high protein burden, as proinsulin biosynthesis has been calculated to reach 10^6^ molecules/min (9). This represents a major challenge for their ER, where protein translation and quality control takes place, and therefore beta cells are particularly susceptible to ER stress (10–12). When faced with ER stress, beta cells respond to activating the unfolded protein response (UPR) (13, 14), whose signaling is mediated via three main transmembrane sensors: IRE1α (endoribonuclease inositol requiring protein), PERK (protein kinase RNA-like endoplasmic reticulum kinase), and ATF6 (activating transcription factor 6) (10–15). In basal conditions, the chaperone immunoglobulin heavy chain binding protein (BiP), a key member of the Hsp70 family, is constitutively bound to the luminal domain of these three sensors and prevents their activation. When misfolded protein accumulates in the ER, BiP dissociates from the UPR sensors leading to their consequent activation. While a moderate ER stress-induced UPR represents a compensatory mechanism, a chronic or overwhelming ER stress impairs cellular functions and can induce apoptosis to remove irreversibly damaged cells (11–15).

Autophagy has been shown to have a protective role against ER stress (16) and facilitate mitochondrial turnover (17). Indeed, it has been demonstrated that beta-cell Atg7^−/−^ mice are characterized by islet degeneration, impaired insulin secretion, and glucose intolerance (18, 19). In addition, we have previously shown that a proportion of beta cells of type 2 diabetic (T2D) subjects presents a major increase of autophagic vacuoles and autophagosomes, associated with cell damage, which further suggests that altered autophagy might contribute to the loss of beta cell functional mass (20).

To shed further light on these issues, we presently explored the effects of autophagy modulation in isolated human islets under conditions of metabolically (palmitic acid) or chemically (brefeldin A) induced ER stress (21, 22). In addition, activators and inhibitors of autophagy were tested with pancreatic islets from T2D organ donors.

## Materials and Methods

### Human Islet Isolation and Culture

Human islet collection and handling were approved by the local Ethics Committee. Human pancreatic islets were isolated by enzymatic digestion and density gradient purification from pancreatic samples of non-diabetic (ND; *n* = 17; age 65 ± 21 years; gender: 5 M/12 F; BMI 23.4 ± 3.3 kg/m^2^) and T2D (*n* = 9; age 76 ± 6 years; 4 M/5 F; gender: BMI 25.4 ± 3.7 kg/m^2^) organ donors as detailed elsewhere (23, 24). For the experiments with palmitate, ND islets were cultured for 1–5 days in M199 medium containing 1% BSA with 10 ng/ml rapamycin (autophagy inducer) (25), 5.0 mmol/l 3-methyladenine or 1.0 nmol/l concanamycin-A (autophagy blockers) (25), either in the presence or absence of 0.5 mmol/l palmitate, prepared as previously reported (24, 26). For the experiments with brefeldin A, ND islets were exposed to the autophagy modulators either in the presence or absence of 0.1 ng/ml of this chemical ER stress inducer. The islets prepared from T2D donors were studied after 24 h incubation with M199 medium containing or not 10 ng/ml rapamycin.

### Electron Microscopy Evaluation

Electron microscopy studies were performed on isolated islets as previously described (27). Islets were fixed with 2.5% glutaraldehyde in 0.1 mol/l cacodylate buffer, pH 7.4 for 1 h at 4°C. After rinsing in cacodylate buffer, the sample was postfixed in 1% cacodylate buffered osmium tetroxide for 2 h at room temperature, then dehydrated in a graded series of ethanol, briefly transferred to propylene oxide and embedded in Epon-Araldite. Ultrathin sections (60–80 nm thick) were cut with a diamond knife, placed on formvar-coated copper grids (200 mesh), and stained with uranyl acetate and lead citrate. The presence of marked chromatin condensation and/or blebs was considered as signs of apoptosis (28). Morphometric analyses were performed by stereological techniques (19, 24, 27). In particular, volume density of insulin granules, mitochondria and rough endoplasmic reticulum (RER) was estimated. Micrographs, obtained at 10,000 × were analyzed by overlay with a graticule (11 × 11 cm) composed of 169 points. Volume density was calculated according to the formula: VD = Pi/Pt, where Pi is the number of points within the subcellular component and Pt is the total number of points, and expressed in ml/100 ml of tissue (ml%) (19, 24, 27).

### Insulin Secretion

Insulin secretion studies were performed by the batch incubation technique as previously described (29–31). Groups of approximately 15 islets of comparable size were incubated at 37°C for 45 min in Krebs-Ringer bicarbonate solution (KRB), 0.5% albumin, pH 7.4, containing 3.3 mmol/l glucose. Then, the medium was removed and replaced with KRB containing 16.7 mmol/l glucose. After additional 45 min incubation, medium was collected. Insulin levels were measured by a commercially available immunoradiometric assay (Pantec Forniture Biomediche, Turin, Italy). Insulin secretion was expressed as stimulation index (SI), i.e., the ratio of stimulated (16.7 mmol/l glucose) over basal (3.3 mmol/l glucose) insulin secretion (29–31).

### Quantitative RT-PCR Experiments in Isolated Islets

Gene expression studies were performed as previously detailed (30). Total RNA was extracted using the PureLink™ RNA Mini kit (Life technologies, Carlsbad, CA, USA) according to manufacturer recommendations and quantified by absorbance at A260/A280 nm (ratio >1.95) in a Nanodrop 2000c spectrophotometer (Thermo Scientific, Waltham, MA, USA). After it was reverse-transcribed with SuperScript VILO Master Mix (Life technologies), the levels of the genes of interest were normalized for the housekeeping gene beta actin and quantified by the 2^−ΔΔ*Ct*^ method in a VIIA7 instrument (Life technologies). The primers/ probe for the analyzed genes were purchased from Taqman® Assay on-demand library (Life technologies).

### p62 Evaluation in Isolated Human Islets

The levels of p62 were assessed in T2D islets by the *p62 Elisa kit* (Enzo Life Sciences, Lausen, Switzerland) following the manufacturer protocol. In brief, after 24 h exposure to rapamycin or 3-MA, the islets were collected, protein were extracted and aliquoted on a plate pre-coated with a p62 specific antibody. After having incubated the samples in presence of a second anti-p62 antibody (rabbit polyclonal), the amount of p62 was revealed by adding a secondary donkey anti-rabbit IgG antibody conjugated to horseradish peroxidase and a mix composed by TMB and hydrogen peroxide. The plate was read in a FLUOstar Omega plate reader (BMG Labtech, Ortenberg, Germany) and the amount of p62 normalized for the total amount of proteins.

### Statistical Analysis

Data are presented as mean ± SD. Differences between groups were assessed by the two-tailed Student's *t*-test or one-way ANOVA followed by the Bonferroni correction, as appropriate. A *P* < 0.05 was considered statistically significant.

## Results

### Beta Cell Apoptosis and Insulin Secretion

**Figure 1:**
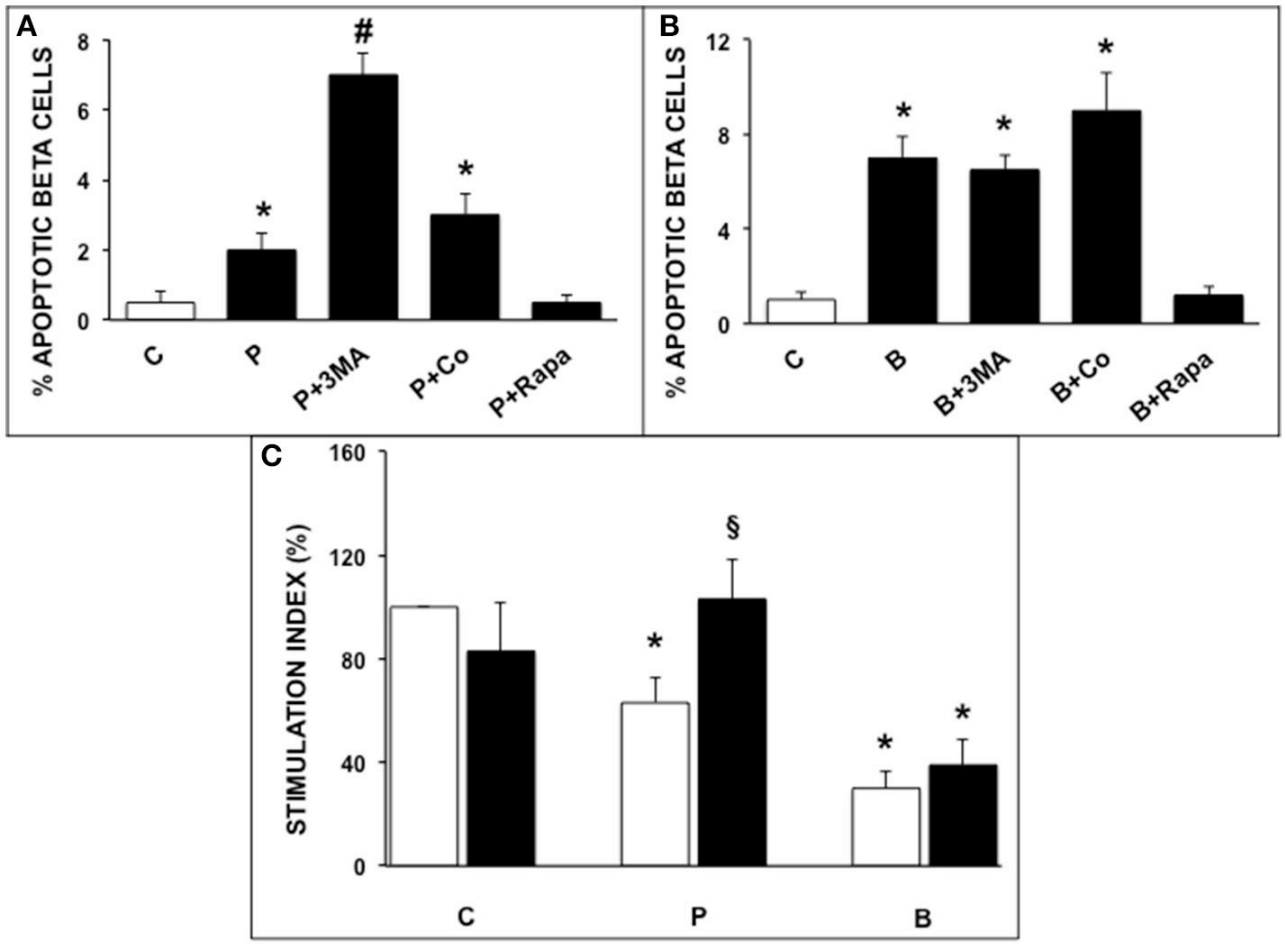
**(A,B)** Ultrastructural morphometric analysis of beta-cell apoptosis in human islets after 5 days exposure to palmitate **(A)** or 1 day exposure to brefeldin **(B)**. C, control; P = 0.5 mmol/l palmitate; B = 0.1 ng/ml brefeldin; P + 3MA = palmitate + 5 mmol/l 3-methyladenine; P + Co = palmitate+ 1 nmol/l concanamycinA; P + Rapa = palmitate + 10 ng/ml rapamycin; B + Rapa = brefeldin + 10 ng/ml rapamycin. For palmitate experiments around 200–300 cells were considered, corresponding to 6 islets and two islet preparations. For brefeldin around 200 cells were analyzed corresponding to 3 islets and 1 islet preparation. Statistical analysis: ^*^p < 0.05 vs. C and P + Rapa or B + Rapa, ^#^*p* < 0.05 vs. all groups, after Bonferroni correction. **(C)** Glucose-stimulated insulin secretion of control (C), palmitate-treated (P), and brefeldin-treated (B) human islets in the presence (black bars) or absence (white bars) of 10 ng/ml rapamycin (*N* = 4–7). Statistical analysis: ^*^p < 0.05 vs. C; ^§^*p* < 0.05 vs. P, after Bonferroni correction.

Exposure of ND human islets to 0.5 mmol/l palmitate for 5 days or 0.1 ng/ml brefeldin-A for 24 h induced a significant increase of beta cell apoptosis compared to control islets (Figures 1A,B), confirming previously reported results (32, 33). In agreement with previous reports (20, 24) apoptosis was identified on the basis of internationally acknowledged criteria based on the appearance of marked chromatin condensation and blebs (28). Electron microscopy can be considered one of the best methods to identify apoptotic cells, because it enables not only the detection of apoptosis but also enables to identify which type of cell is undergoing apoptosis (see also Figure S1 ESM). The deleterious effects of both ER stressors were prevented by rapamycin (autophagy activator), whereas 3-MA, but not concanamycin (both are autophagy inhibitors), enhanced the rate of beta cell apoptosis in presence of palmitate (Figures 1A,B). Insulin secretion was significantly decreased by islet exposure to either palmitate or brefeldin A, and the presence of rapamycin protected beta cells from palmitate-induced insulin secretion alterations (Figure 1C).

### Electron Microscopy Studies

**Figure 2:**
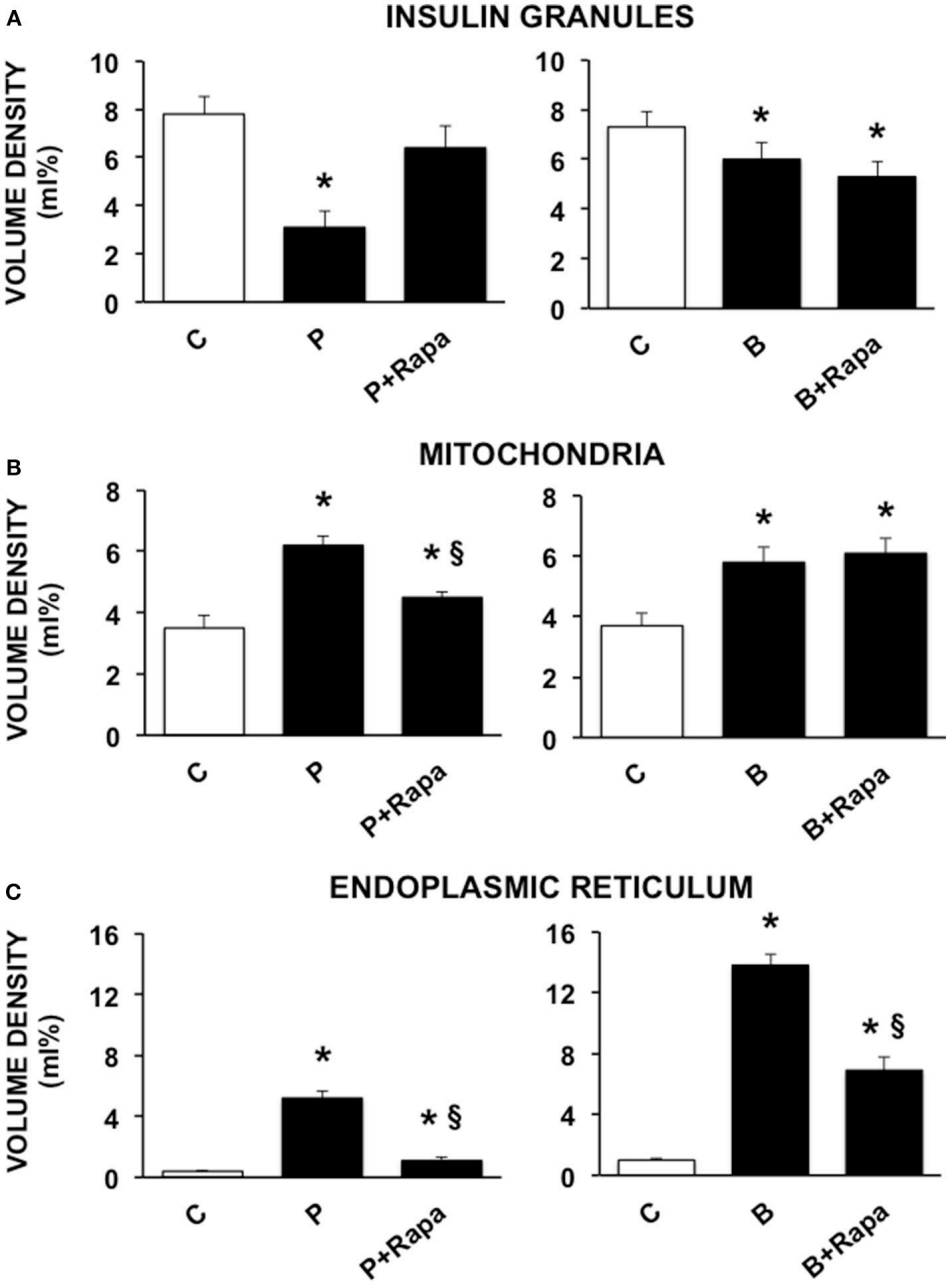
Quantitative morphometric analysis of volume density of insulin granules **(A)**, mitochondria **(B)**, and endoplasmic reticulum **(C)** in beta cells of isolated human islets after 5 days exposure to palmitate (left panels) or 1 day exposure to brefeldin (right panels). C, control; P = 0.5 mmol/l palmitate; B = 0.1 ng/ml brefeldin; P + Rapa = palmitate + 10 ng/ml rapamycin; B + Rapa = brefeldin + 10 ng/ml rapamycin. For palmitate experiments around 200–300 cells were considered, corresponding to 6 islets and two islet preparations. For brefeldin around 200 cells were analyzed corresponding to 3 islets and 1 islet preparation. Statistical analysis: ^*^p < 0.05 vs. C; ^§^*p* < 0.05 vs. P, after Bonferroni correction.

We then investigated the ultrastructural changes induced by the exposure of human islets to palmitate or brefeldin by quantitative morphometry. In particular, we assayed the volume density of insulin granules, mitochondria, and ER in beta cells exposed to 0.5 mmol/l palmitate for 5 days and 0.1 ng/ml brefeldin for 24 h. In addition, we evaluated the effects of the stimulation of autophagy by the concomitant exposure to 10 ng/ml rapamycin. Five days of palmitate exposure significantly decreased the volume density of insulin granules, whereas volume density of both mitochondria and ER was significantly increased (Figures 2A–C). In all cases, co-incubation with rapamycin was able to prevent the changes caused by palmitate exposure (Figures 2A–C). Brefeldin A also induced a significant reduction of insulin granule volume density, although quantitatively less compared to palmitate, and both mitochondria and ER volume density was significantly increased (Figures 2A–C). Rapamycin had no effects on the changes in insulin granules and mitochondria induced by brefeldin, whereas it was able to partially prevent brefeldin A-induced increase of ER volume density (Figures 2A–C).

### Gene Expression in Isolated Human Islets

**Figure 3:**
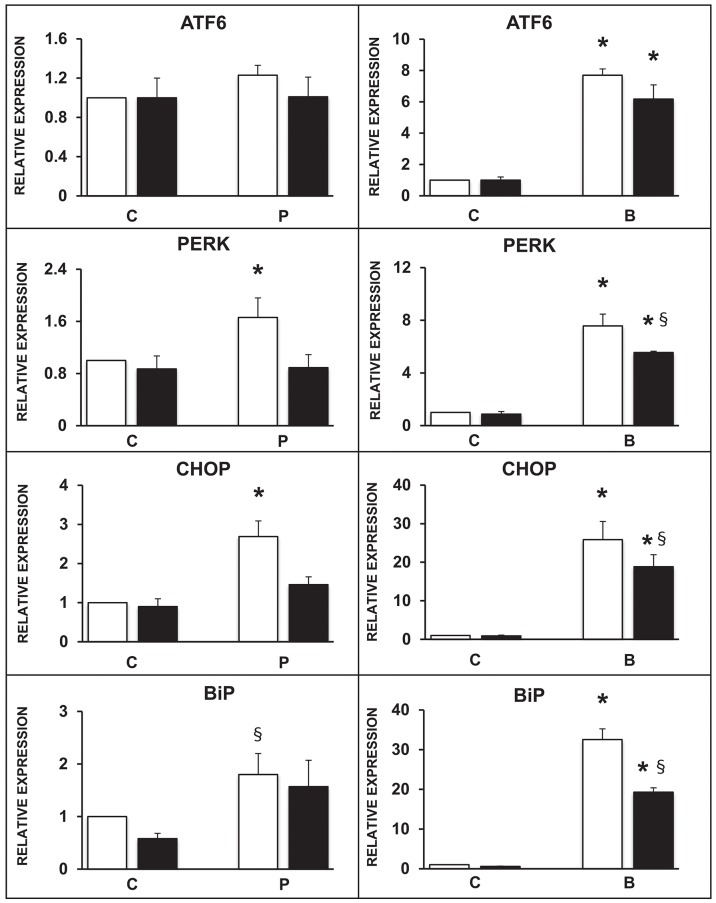
Expression of several ER stress markers in isolated human islets after 5 days exposure to palmitate (left panels) or 1 day exposure to brefeldin (right panels) in the presence (black bars) or absence (white bars) of 10 ng/ml rapamycin. *N* = 4–5 for both treatments. C, control; P = 0.5 mmol/l palmitate; B = 0.1 ng/ml brefeldin; Statistical analysis: ^*^p < 0.05 vs. all the other groups; ^§^*p* < 0.05 vs. C, after Bonferroni correction.

The expression of selected ER markers was then studied in isolated ND human islets exposed for 24 h to 0.5 mmol/l palmitate or to 0.1 ng/ml brefeldin; the modulating effect of the concomitant exposure to 10 ng/ml rapamycin was also evaluated. Palmitate exposure significantly increased the expression of PERK, CHOP, and BiP with respect to control human islets, and this effect was significantly prevented by the concomitant presence of rapamycin (Figure 3, panels on the left). Brefeldin-A exposure markedly increased the expression of all the assayed ER stress markers in human islets with respect to controls, and the concomitant stimulation of autophagy by rapamycin in part counteracted this effect (Figure 3, panels on the right).

### Rapamycin and T2D Human Islets

**Figure 4:**
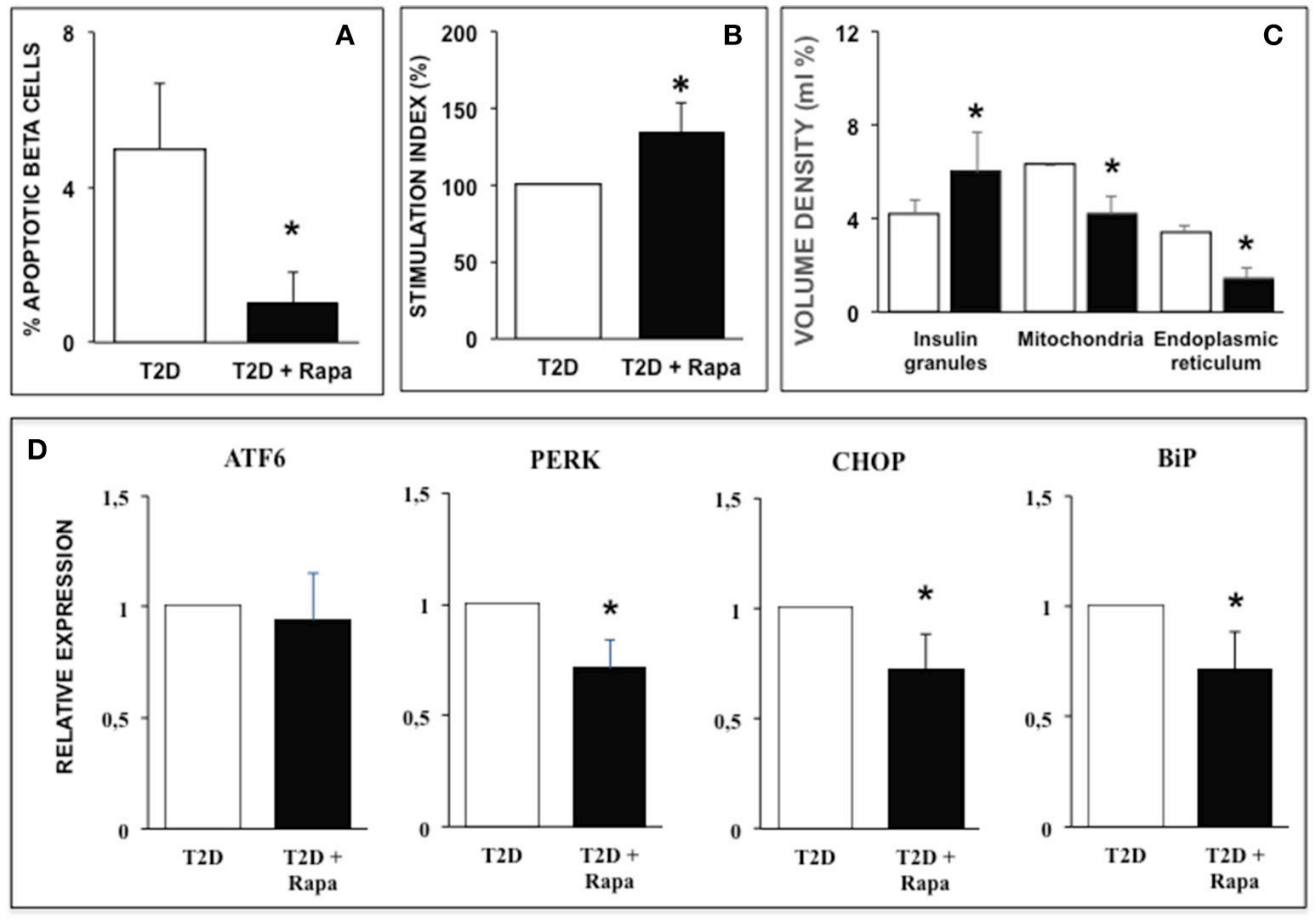
**(A)** Ultrastructural morphometric analysis of beta-cell death in T2D human islets in the presence (black bar) or absence (white bar) of 10 ng/ml rapamycin. *N* = 9 (300–400 cells, two islet preparations analyzed). Statistical analysis: ^*^p < 0.05 vs. T2D. **(B)** Glucose-stimulated insulin secretion of type 2 diabetes human islets in the presence (black bar) or absence (white bar) of 10 ng/ml rapamycin. *N* = 5. Statistical analysis: ^*^p < 0.05 vs. T2D. **(C)** Quantitative morphometric analysis of volume density of insulin granules, mitochondria, and endoplasmic reticulum in beta cells of type 2 diabetes human islets in the presence (black bar) or absence (white bar) of 10 ng/ml rapamycin. *N* = 9 (300–400 cells, two islet preparations analyzed). Statistical analysis: ^*^p < 0.05 vs. T2D. **(D)** Expression of several ER stress markers in type 2 diabetes human islets in the presence (black bars) or absence (white bar) of 10 ng/ml rapamycin. *N* = 5. Statistical analysis: ^*^p < 0.05 vs. T2D.

Finally, we explored the effects of rapamycin in human islets isolated from T2D donors. In T2D islets beta cell apoptosis was significantly higher than in ND islets, in agreement with previous results (31, 34), and exposure to rapamycin significantly decreased the number of apoptotic beta cells (Figure 4A). Rapamycin also caused a significant increase of glucose-stimulated insulin secretion T2D human islets (Figure 4B).

Quantitative morphometry confirmed the ultrastructural alterations previously observed in human islets isolated from T2D patients (31, 34): volume density of insulin granules was significantly decreased whereas volume density of both mitochondria and ER was significantly increased compared to ND islets (Figure 4C). Exposure of diabetic islets to rapamycin was able to significantly counteract these alterations (Figure 4C). These favorable changes induced by rapamycin were associated with decreased expression of PERK, CHOP, and BiP similar to those observed in ND islets exposed to palmitate (Figure 4D).

### p62 in T2D Islets

**Figure 5:**
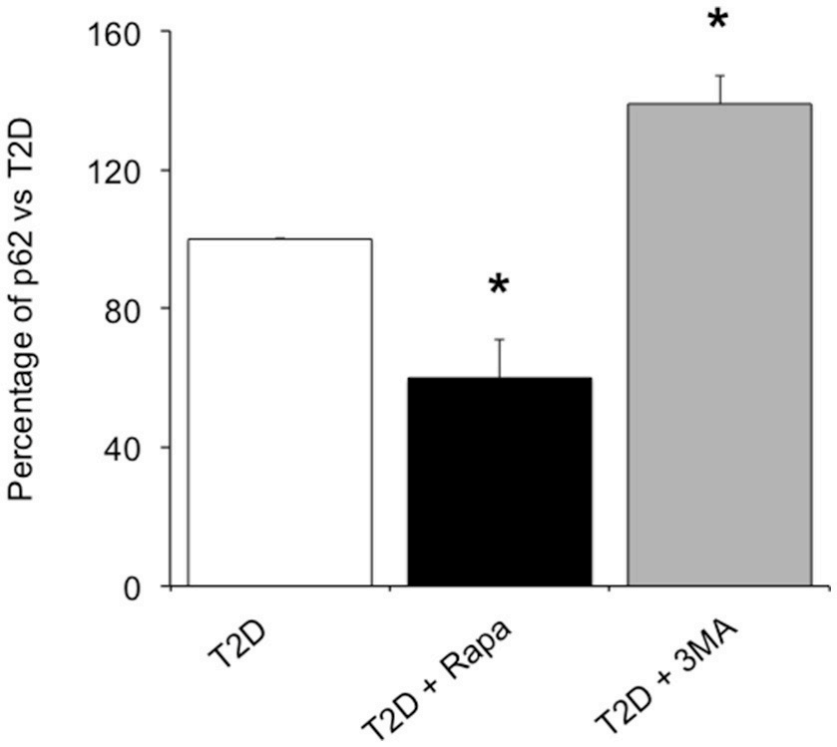
Levels of p62 in T2D human islets in presence of 10 ng/ml of the autophagy inducer, rapamycin, or 5.0 mmol/l of the autophagy inhibitor, 3-MA. *N* = 3–4. Statistical analysis: ^*^p < 0.05 vs. all groups.

In order to confirm whether rapamycin and 3-MA could modulate autophagy flux, we evaluated the amount of p62, an autophagy receptor that accumulates in cells when macroautophagy is inhibited (21). In ND islets, we found 1.15 ± 0.36 ng/ml of p62/μg total proteins. In T2D islets, p62 levels (3.62 ± 0.90 ng/ml of p62/μg total proteins) were higher with respect to non-diabetic islets (*p* < 0.05). Exposure to rapamycin significantly reduced the amount of p62 (Figure 5), indicating that the drug could increase the autophagy flux in T2D islets. Conversely, the use of 3-MA, an autophagy inhibitor, significantly increased the levels of the receptor (Figure 5).

## Discussion

The synthesis, modification and delivery of proteins to their target sites occur in the ER. Several physiological and pathological conditions are able to impair these processes leading to ER stress, and in the last years a large body of evidences has been provided indicating that ER stress may be implicated in beta cell dysfunction and death in diabetes (22, 23, 35–42).

Recently, autophagy has emerged as a crucial protective mechanism during ER stress (43, 44). An early study reported that cells undergoing ER stress, as indicated by ER expansion, showed a concomitant increase in autophagosome abundance (44). This activation of autophagy was interpreted as a survival mechanism to prevent ER stress-induced toxicity (43, 44). In mammals, ER stress inducers generally act as on-off switches for mTOR-regulated cell growth, survival and energy balance, through the downregulation of AKT1, which induces the activation of autophagy by decreasing mTOR activity (45). Autophagy is a degradation mechanism that can be induced by starvation or other form of nutrient deprivation to supply substrates for cellular energy generation (46). Autophagy also serves as a catabolic pathway to recycle excessive or damaged intracellular organelles such as mitochondria (47). Therefore, it can act as a housekeeping mechanism in the absence of stress, while under stress conditions it exerts a crucial protective role (48).

The aim of this study was to explore the effects of the modulation of autophagy on the ER stress-induced beta-cell dysfunction in isolated human pancreatic islets. We induced ER stress in cultured human islets by their exposure to a metabolic (0.5 mmol/l palmitic acid for 5 days) or a chemical (0.1 ng/ml brefeldin for 1 day) stressor. The increased levels of fatty acids, commonly associated with obesity, can induce insulin resistance and beta-cell dysfunction, making them good candidates to explain the link between obesity and the development of T2D (49–51). It has been proposed that palmitate-induced ER stress may arise from the disruption of protein processing and trafficking (49), or from incorrect Ca^++^ regulation (52). Brefeldin A is a macrocyclic lactone antibiotic which is synthetized from palmitate by several fungi and has been shown to induce ER stress through the inhibition of ADP ribosylation factor (ARF) and the consequent disruption of the ER-Golgi vesicular transport (53, 54).

Here, we confirm that lipotoxic conditions (such as palmitate exposure) and chemically-induced (brefeldin) ER stress are associated with alterations of beta cell survival and function, and show that the modulation of autophagy influences these effects. In particular, rapamycin, an inducer of autophagy through inhibition of mTORC1 complex (25), was able to prevent beta cell apoptosis induced by palmitate or brefeldin, and restore a proper insulin secretion in response to glucose which was altered by the presence of the fatty acid. These data are in line with those previously published on the effect of rapamycin in *Akita* mice (55), where the drug attenuated cellular stress and apoptosis that, conversely, were exacerbated by autophagy inhibitors in conditions of accumulation of misfolded proinsulin. Conversely, 3-MA and concanamycin-A were used as autophagy inhibitors. The first blocks an early stage of autophagy by inhibiting the class III PI3K (21), while concanamycin causes an increase in lysosomal/vacuolar pH, and, ultimately, blocks fusion of autophagosomes with the vacuole by inhibiting V-ATPase (25). In our study, the presence of 3-MA caused a significant increase of beta-cell apoptosis in human islets exposed to palmitate, whereas concanamycin A did not show any effect on the cytotoxicity induced by palmitate or brefeldin. Our data are in agreement with previously published results in other cell types (56), where it was observed that inhibition of autophagy at an early stage, but not at a late stage, potentiated chemosensitivity, increasing caspase 3/7 activation, especially in conditions of high levels of autophagy. More in detail, 3-MA could exert this pro-apoptotic action making available beclin-1 (a master component of the PI3K-III complex inhibited by the drug, playing a role also in apoptosis) for caspase 8 cleavage in order to elicit cell death (57–60). However, other studies exist reporting a more deleterious effect of late autophagy blockade on cell survival (61).

At the ultrastructural level, we found that in both palmitate and brefeldin-treated human islets a significant decrease in the volume density of insulin granules, and a significant increase in volume density of mitochondria and ER was present. Co-exposure with rapamycin was able to prevent these alterations mainly in the islets treated with palmitate. These beneficial effects on ultrastructure were associated with a reduction in the expression of some ER stress-related genes. The ultrastructural alterations found in our experiments were similar to those previously observed in other studies. In particular, they look like those observed in beta cells isolated from Atg^Δβ−*cell*^ mice with beta cell-specific deletion of Atg7 (autophagy-related 7) (19) indicating that autophagy could be necessary to maintain beta-cell homeostasis (62, 63) and in human islets isolated from T2D patients (20, 23, 27) confirming a pathogenetic role played by ER stress (50, 64). However, the role of autophagy in diabetes pathophysiology has not been fully elucidated. Recently, it was suggested that impaired autophagy could lead to accumulation of dysfunctional organelles such as mitochondria (65) and that in type 2 diabetic pancreatic beta cells, a massive overload of autophagic vacuoles and autophagosomes might contribute to the loss of beta-cell mass (20). In addition, some authors reported that rapamycin improved insulin resistance and hepatic steatosis in T2D rats via activation of autophagy (66). Our results in T2D islets showed that the promotion of autophagic process by rapamycin, as evaluated by the clearance of the p62 protein, is associated with amelioration of function, survival and ultrastructure possibly due to reduction of ER stress. Recently, it was hypothesized that mTORC1 (one of the major signaling complex in beta cells, where it is responsible for nutrient sensing and beta cell growth) (67), if short term and transiently activated, regulates beta cell replication, anabolic growth and insulin secretion under physiological conditions (67). Conversely, its sustained activation (as in presence of chronic excess of nutrients) is associated with impaired insulin release, ER stress and reduced beta cell survival. In this regards, several authors showed that chronic exposure to high glucose and/or high free fatty acid concentrations could activate mTORC1 in beta cells and that its inhibition could prevent gluco- lipotoxic-induced beta cell derangement (68). In particular, Yuan and colleagues (69) showed that islets isolated from T2D organ donors had increased mTORC1 activity in comparison with non-diabetic islets and that this augmentation was present in beta but not alpha cells. Moreover, mTORC1 genetic or chemical inhibition was associated with restoration of insulin release in T2D islets. Differently from them, in our study, 10 ng/ml rapamycin (corresponding to around 11 nmol/l) were able to promote beta cell function, survival and ultrastructure in T2D islets. Similarly, it has been observed that the defective autophagic flux associated with a lysosomal dysfunction observed in T2D islets can be restored also by a GLP-1 receptor agonist (70). It should be underlined that the beneficial effects observed with rapamycin in presence of ER stress modulators, especially those related to insulin release, are exerted in a short-term setting. Additional experiments should be performed to assess the role of a chronic exposure to the drug. In this regards, some authors have observed how some pharmacological agents, currently used in diabetes treatment, show deleterious effects in beta cells when chronically administered in presence of rapamycin (71).

In conclusion, this study provides information on how conditions of metabolically or chemically induced stress on human beta cells associate with reduced beta cell survival, impaired beta cell function, and ultrastructural alterations, which are also mediated by ER stress. More importantly, our data suggest that promotion of autophagy at the beta cell level, in some context, might be helpful to protect beta cell health.

## Ethics Statement

The study was approved by the Ethics Committee of the University of Pisa.

## Author Contributions

MB, PM, and VD conceived the study and wrote the manuscript. MB, SM, FG, MS, LM, UB, PD, DE, and MC researched data. All the authors contributed to discussion and reviewed the manuscript. VD is the guarantor of this study.

### Conflict of Interest Statement

The authors declare that the research was conducted in the absence of any commercial or financial relationships that could be construed as a potential conflict of interest.
