# Supplementary figures and images for: Modulation of Autophagy Influences the Function and Survival of Human Pancreatic Beta Cells Under Endoplasmic Reticulum Stress Conditions and in Type 2 Diabetes
Source: Front Endocrinol (Lausanne). 2019 Feb 26;10:52. doi: 10.3389/fendo.2019.00052 (PMC6399112; doi:10.3389/fendo.2019.00052)

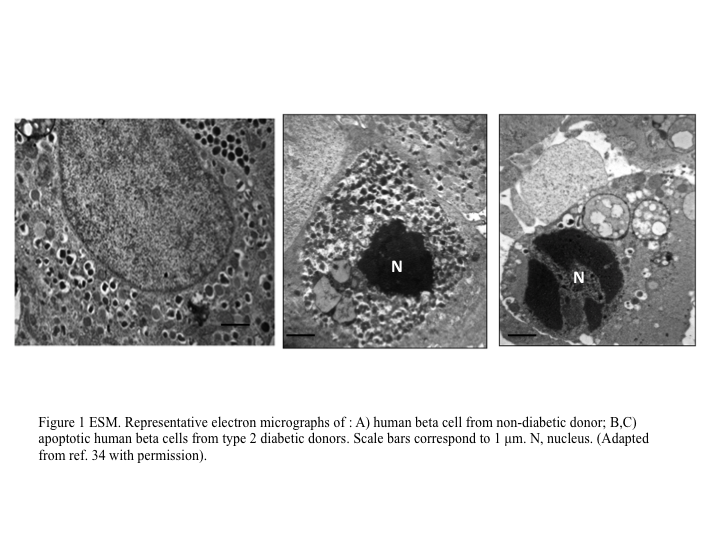

Supplement: Supplementary file 1 [file Image_1.TIFF]
